# Rethinking directiveness in AI coaching chatbots

**DOI:** 10.3389/fpsyg.2026.1822088

**Published:** 2026-06-23

**Authors:** Nicky H. D. Terblanche, Andrew Beric Brown

**Affiliations:** Stellenbosch Business School, Stellenbosch University, Cape Town, South Africa

**Keywords:** AI coaching, AI Device Use Acceptance (AIDUA), Big Five personality traits, directive coaching, generative AI, goal attainment, non-directive coaching, technology adoption

## Abstract

Organizational coaching is well established as an effective intervention for professional and personal development. However, ongoing debate exists regarding the relative merits of directive versus non-directive coaching styles, with non-directive approaches widely accepted as desired practice in human-to-human coaching. Recent advances in generative artificial intelligence (Gen AI), alongside the widespread adoption of applications such as ChatGPT, have created the conditions for Gen AI-powered coaching chatbots to emerge as potential coaching alternatives, with initial studies showing promising results. The advent of these coaching chatbots raise many questions including to what extent they should mimic what human coaches do, including their level of directiveness. To investigate this knowlede gap, two Gen AI coaching chatbots, one directive and one non-directive, were developed using Heron’s intervention analysis model to operationalise distinct coaching styles. A between-subjects quasi-experiment (*n =* 158) measured and compared these AI coaching style preferences across four dimensions: technology adoption, working alliance, goal attainment, and personality traits. Contrary to established coaching conventions, results revealed a higher rating for the directive coaching style with respect to technology performance expectancy, working alliance, and goal attainment. Users exhibiting high levels of extraversion, conscientiousness, and openness to experience rated the directive approach higher. These findings raise questions about the prevailing non-directive coaching paradigm in AI coaching contexts and suggest that Gen AI coaching chatbots for workplace use should incorporate directive features tailored to coachee personality. The implications for coaching practice, AI coaching design, and future research are discussed.

## Introduction

The world of organizational coaching has recently been disrupted by Gen AI coaching chatbots, which have demonstrated positive goal-based outcomes across several studies (Graßmann et al., 2020; Terblanche et al., 2022; Terblanche, 2024b). Yet a fundamental question remains unresolved: what level of directiveness do users prefer when engaging with an AI coaching chatbot? The debate between directive and non-directive coaching styles is longstanding (Ives, 2008). A directive style focuses on guiding coachee behaviour, sharing knowledge, and challenging assumptions, whereas a non-directive style is facilitative, encouraging self-discovery and autonomous learning (De Haan and Nilsson, 2017; Ives, 2008). While the International Coaching Federation advocates predominantly non-directive practice (Updated ICF Core Competencies, 2019), some evidence from human-to-human coaching suggests that coachees prefer a more directive style in certain circumstances (Cavanagh, 2006; Stober and Grant, 2010; Terblanche, 2021; Wood, 2015).

Gen AI entered the scene with the launch of ChatGPT in November 2022, attracting over 100 million users within two months (Peres et al., 2023). Currently the most prevalent use cases for Gen AI applications centre on professional support, personal development, and learning (Zao-Sanders, 2025). This type of development has long been associated with organizational coaching, which has a well-established evidence base for supporting both professional and personal development (Theeboom et al., 2014). These developmental domains also align directly with a specific generation of users, the millennials. Although all generations value growth and self-improvement, millennials are particularly strongly orientated toward self development. Millennials in particular have been shown to respond positively to coaching as a developmental intervention (Solomon and Van Coller-Peter, 2019).

The millennial generation, or Generation Y, comprises individuals born between 1980 and 1999 who began entering the workforce in the early 2000s (Sujansky and Ferri-Reed, 2009). Millennials are now the dominant generational cohort in the global workforce constituting approximately 75% of the working population (Timmes, 2022). Millennials are characterised by their technological proficiency, appetite for purpose-driven work, and commitment to continuous learning (Twenge et al., 2010; Waleed et al., 2023). Notably, 22% report using AI in the workplace for all or most of their working time (Deloitte, 2024).

The question of directive versus non-directive coaching takes on additional complexity in the context of AI. Popular Gen AI applications such as ChatGPT, Gemini, and LLaMA operate in a fundamentally directive manner where users ask questions and receive answers (Bommasani et al., 2021). This transactional dynamic may shape millennial expectations when engaging with an AI coaching chatbot, potentially shifting their preferences away from the non-directive paradigm associated with human coaching.

Research suggests that coachee personality traits can impact coaching success (Stewart et al., 2008). This raises the question of whether individual differences in coachee personality could impact preferences for directive or non-directive AI coaching chatbot styles.

This study addresses these gaps by examining user preferences for directive versus non-directive coaching styles in Gen AI chatbots. We ask the following research questions in relation to millennial users:

1. What are the differences in technology adoption between a directive vs. non-directive AI coaching chatbot?
2. What are the differences in working alliance between a directive vs. non-directive AI coaching chatbot?
3. What are the differences in goal attainment between a directive vs. non-directive AI coaching chatbot?
4. What is the role of personality traits of millennials in their technology adaption, working alliance and goal attainment when using a directive vs. non-directive AI coaching chatbot?

Accordingly, the objectives of this study are to examine differences in technology adoption, working alliance, and goal attainment between directive and non-directive AI coaching chatbots, and to assess the moderating role of personality traits in these relationships. Addressing these questions could create a better understanding of how these two different coaching approaches influence user experience and outcomes of AI coaching chatbots. Specifically, the study contributes to the body of knowledge by examining whether established assumptions in human coaching favouring non-directive coaching extend to AI, These insights could offering insights to designers of AI coaching chatbots for the development and deployment of AI coaching systems.

## Literature review

### Directive versus non-directive coaching styles

The debate between directive and non-directive coaching styles is longstanding and unresolved (De Haan and Nilsson, 2017; Ives, 2008; Parsloe and Leedham, 2016; Wood, 2015). Historically, coaching was directive in nature, involving guidance, instruction, and knowledge transfer, drawing on the traditions of sports coaching and mentoring (Parsloe and Leedham, 2016). As the discipline matured, it was heavily influenced by Carl Rogers’ non-directive client-centred therapy (Rogers, 1951), shifting the dominant view toward facilitative, coachee-led approaches (Bluckert, 2006). The International Coaching Federation now advocates predominantly non-directive practice as the professional standard (Updated ICF Core Competencies, 2019). A directive coaching style focuses on guiding coachee behaviour, sharing knowledge, and challenging assumptions, whereas a non-directive style is facilitative, supporting self-discovery and autonomous learning (De Haan and Nilsson, 2017; Ives, 2008).

However, the evidence base for the primacy of non-directive coaching in practice is more nuanced than the professional standards suggest. Parsloe and Leedham (2016) propose a continuum, arguing that the appropriate style depends on the objective, context, and duration of the coaching engagement. Grant (2014b) concurs, noting that the core question is not whether directive or non-directive approaches are correct, but which approach most effectively supports the coachee in achieving their goals at any given moment. A skilled coach navigates fluidly between directive and non-directive modes as the situation demands. Wood (2015) found that providing ideas and introducing frameworks or perspectives into the coaching conversation, is a common and valued practice among professional coaches, despite its apparent tension with strict non-directive principles. Cognitive load theory further supports the value of directive input, proposing that individuals with limited domain knowledge benefit from structured guidance and worked examples to reduce cognitive demand and facilitate effective problem-solving (Atkinson et al., 2000; Sweller, 1988). The debate continues.

**Figure 1:**
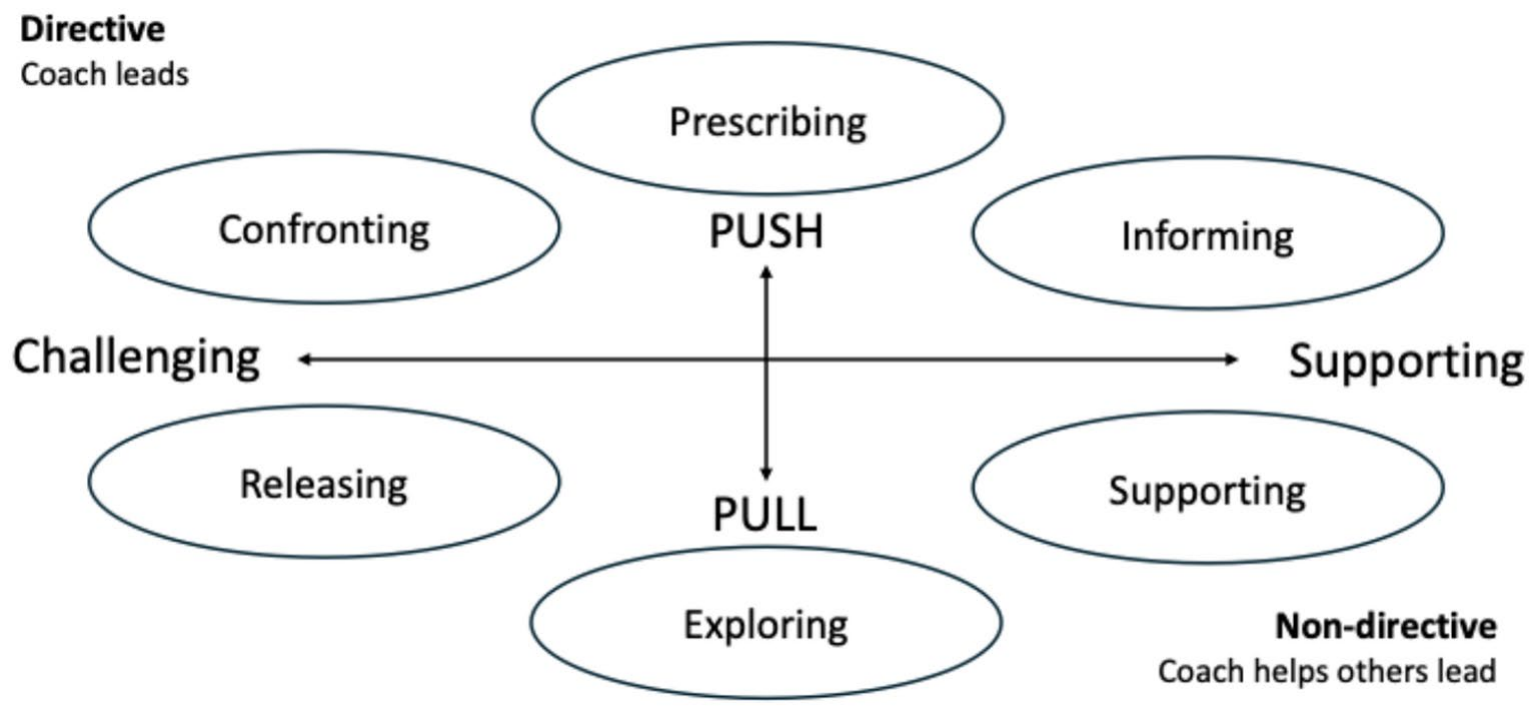
The “Heron model” (De Haan and Nilsson, 2017).

A useful lens to classify directive versus non-directive communication and coaching is Heron (2001) six-category intervention analysis model (Figure 1). Originally developed to improve understanding of interpersonal relations in personal development contexts, the model classifies one-on-one interventions along two axes: directive (push) versus non-directive (pull), and challenging versus supportive (De Haan and Burger, 2014). Directive interventions comprise three categories: prescribing (providing direction, advice, and recommendations), informing (sharing knowledge and professional feedback), and confronting (challenging assumptions and stimulating awareness of behaviours or beliefs). Non-directive interventions similarly comprise three categories: releasing (helping the coachee manage emotions that block progress), exploring (facilitating self-discovery through active listening and open questioning), and supporting (enhancing coachee confidence, self-esteem, and motivation) (De Haan and Nilsson, 2017; Heron, 2001). Heron (2001) himself advocates for balance between these approaches, noting that an overreliance on either directive or non-directive interventions diminishes coaching effectiveness, and that skilled practitioners adapt their style according to context, coachee, and objective.

The Heron model has been applied across healthcare, education, and coaching research, and De Haan and Nilsson (2017) have identified its intervention categories as representing the “common factors” that coachees experience as the full range of coaching techniques, factors that are also central to the working alliance between coach and coachee.

Empirical evidence on coachee style preferences is revealing. De Haan and Nilsson (2017) surveyed 1,302 coaches and coachees using a Heron-based instrument and found that coachees consistently rated their coaches as more directive than coaches rated themselves, suggesting an appetite for directive input that practitioners may systematically underestimate. Terblanche (2021) found that managers undergoing career transitions particularly valued coaches who shared knowledge through frameworks and offered direct challenge, while also appreciating non-directive questioning and reflection, reinforcing the case for contextual flexibility rather than ideological adherence to either style.

It seems that there is an ongoing tension in human-to-human coaching about the level of directiveness. The introduction of Gen AI coaching chatbots further exasperates this debate especially when focussing on the Millennial generation who are tech-savvy, uses Gen AI reegularly and are the most represented in the current workforce. These concepts will be explored next.

### Generative AI and coaching chatbots

Gen AI refers to machine systems capable of perceiving, reasoning, learning, and generating content in response to natural language prompts (Rai et al., 2019). The release of ChatGPT in November 2022 marked a turning point in mainstream AI adoption, attracting over 100 million users within two months (Peres et al., 2023) and exceeding 400 million weekly active users by February 2025 (DemandSage, 2025). Other significant platforms include Google Gemini, Meta’s LLaMA-powered applications, and Perplexity AI (Gartner, 2025). By 2025, 78% of organisations globally reported regular use of Gen AI, nearly doubling from the prior year (McKinsey, 2025). A 2025 Salesforce survey found that 65% of Gen AI users are millennials or Generation Z, and 72% are employed (SalesForce, 2025). Analysis of the top Gen AI use cases in 2025 reveals a pronounced shift toward personal and professional support, with learning and development ranked fourth (Zao-Sanders, 2025) demonstrating patterns directly consistent with millennial motivations for continuous learning and self-improvement (Deloitte, 2024) and the linked to what organizational coaching already offers (Solomon and Van Coller-Peter, 2019).

AI coaching has been defined as “a machine-assisted, systematic process to help coachees set professional goals and construct solutions to efficiently achieve them” (Graßmann and Schermuly, 2021, p. 109). The introduction of AI coaching into the workplace is both inevitable and carries significant potential to scale and democratise coaching as a developmental intervention (Terblanche, 2024a). Research has demonstrated that goal-based AI coaching chatbots can produce outcomes comparable to human coaching in terms of goal attainment (Terblanche et al., 2022), and that they can establish a working alliance with coachees (Graßmann et al., 2020; Terblanche, 2024b). However, important limitations persist. Current AI chatbots lack the capacity to express empathy, demonstrate emotional intelligence, or respond to emotional cues with the flexibility of a skilled human coach, capabilities considered essential for transformative rather than purely performance-based coaching (Passmore and Tee, 2023). Graßmann and Schermuly (2021) suggest that while AI can steer coachees through much of the coaching process, the greatest difficulties lie in identifying coachee-specific problems and providing truly individualised feedback. Bachkirova and Kemp (2025) go further, arguing that AI coaching chatbots fail to meet fundamental coaching criteria and represent an “ersatz” substitute for human coaching, suggesting that AI-facilitated learning may be a more appropriate classification. Despite these critiques, the evidence base for goal-directed AI coaching continues to grow. Ethical challenges, including data privacy, algorithmic bias, confidentiality risks, and the potential for harm, are increasingly being addressed through emerging international regulatory frameworks (Murgia and Espinoza, 2024; Passmore and Tee, 2023).

Critically for this study, research into user expectations of AI chatbots across service, healthcare, and educational contexts consistently reveals positive adoption outcomes when chatbots deliver information and guidance, behaviours that are fundamentally directive in nature (Abd-Alrazaq et al., 2021; Menon and Shilpa, 2023; Uddin et al., 2024). Moore et al. (2017) identify four main conversational types in AI chatbot design: ordinary conversation, service conversation, teaching conversation, and counselling conversation. Service and teaching conversations are directive by nature, while counselling conversations are non-directive. The majority of current AI chatbot research and deployment falls into the service and teaching categories, where users expect and receive answers to their questions. Terblanche and Kidd (2022) confirmed that performance expectancy, the belief that the chatbot will be useful in delivering valued outcomes, is the strongest predictor of adoption intention, a finding corroborated by Van der Merwe and Terblanche (2025) in a study where 67% of participants were millennials. This pattern strongly suggests that millennials, whose AI usage experience is predominantly directive in nature, may bring similar expectations to AI coaching interactions.

### Millennials in the workforce

Millennials were selected as the focal cohort in the present study due to their unique positioning at the intersection of digital fluency and organisational responsibility (Galdames and Guihen, 2022). As mid- to senior-level professionals, they are both recipients and implementers of coaching interventions, making their responses particularly relevant for organisational adoption of AI coaching. Unlike younger cohorts, Millennials are not fully AI-native (Salam et al., 2026), allowing for greater variance in technology acceptance and relational dynamics, which is important for examining how directive and non-directive coaching styles operate within AI coaching.

Generational theory, rooted in Mannheim (1952) foundational essay and subsequently developed by Strauss and Howe (Van Twist et al., 2021), provides a widely accepted mechanism for grouping populations shaped by shared historical and cultural influences. While some scholars caution that generational labels risk perpetuating unfounded generalisations and reinforcing power differentials between age groups (Jauregui et al., 2020), the framework remains a useful lens for understanding cohort-level patterns in behaviour and attitude. The millennial generation, individuals born between the early 1980s and 1999, grew up during a period of rapid technological change, witnessing the rise of the internet, mobile technology, and the globalisation of information, emerging as the first truly digitally native cohort (Waleed et al., 2023). Globally, millennials number approximately 1.8 billion, constituting 23% of the world population (Neufeld, 2021), 21% of the United Kingdom population (StatisticsTimes, 2025), and approximately 75% of the workforce (Timmes, 2022), with many now occupying senior leadership positions (Deloitte, 2016).

Millennials are characterised by their technological proficiency, preference for purpose-driven work, and commitment to continuous learning (Twenge et al., 2010; Waleed et al., 2023). The Deloitte Global 2024 Gen Z and Millennial Survey found that 22% of millennials use AI in the workplace for all or most of the time, with a further 38% using it occasionally (Deloitte, 2024). Respondents who frequently use AI at work reported greater excitement about and trust in the technology, suggesting that familiarity drives positive adoption attitudes. Research confirms that millennials respond positively to coaching as a developmental intervention, particularly in terms of goal identification, organisational engagement, and performance improvement (Solomon and Van Coller-Peter, 2019). Coaching enables millennials to better identify with their organisation’s goals and values, meeting their need for purpose-driven and meaningful work. This combination of coaching receptivity and growing AI adoption makes millennials an instructive population for studying preferences in AI coaching chatbots.

Given this overview, what remains unclear is how directive vs. non-directive coaching style preferences translate to AI coaching contexts, and specifically whether millennials, whose everyday AI experience is predominantly directive, prefer a directive or non-directive coaching style when engaging with an AI coaching chatbot.

## Materials and methods

### Research design

This study employed a between-subjects quasi-experiment design to compare millennial ratings for directive versus non-directive AI coaching chatbots. Two independent groups of participants each engaged with one chatbot condition, completing a survey before and after their AI chatbot coaching session. The independent variable was chatbot coaching styles (directive versus non-directive) and the dependent variables were technology adoption, working alliance, goal attainment, and personality traits.

Although prior coaching literature distinguishes directive and non-directive approaches, there is limited empirical evidence regarding how these approaches are experienced when instantiated in AI coaching chatbots. In addition, competing theoretical perspectives on directive versus non-directive human coaching offer conflicting predictions of application in AI coaching. Given the limited prior empirical research comparing directive and non-directive AI coaching styles, the study adopted an exploratory, research question-driven experimental design intended to support theory development rather than confirmatory hypothesis testing.

### Chatbot design

**Figure 2:**
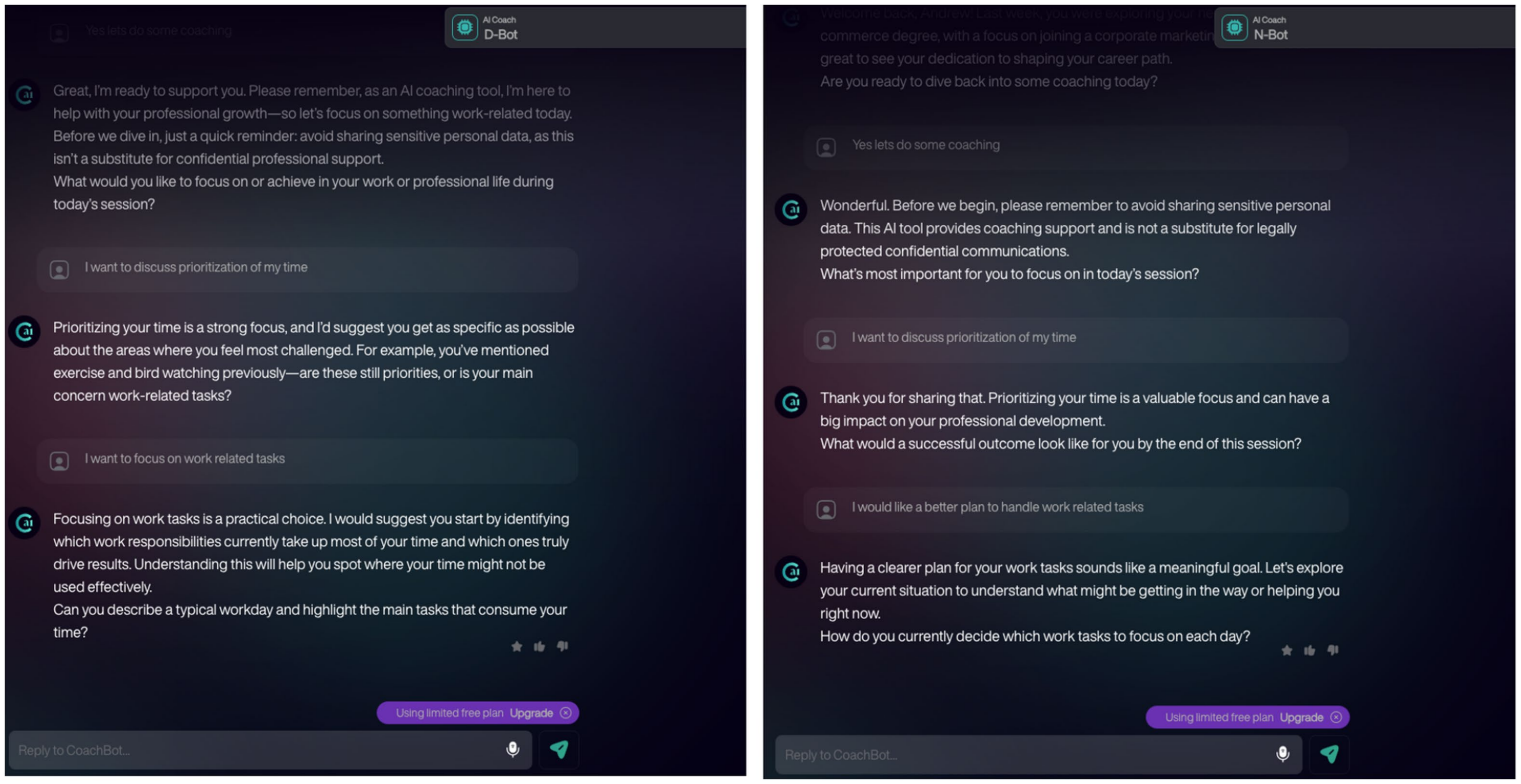
Example conversations with D-Bot (left) and N-Bot (right).

Two web-based coaching chatbots were developed using CoachBot.AI (2026) a secure, GDPR-compliant platform that enables coaches to design and deploy customised coaching chatbots. The platform uses API calls to LLMs such a ChatGPT to generate AI chatbot interaction conversations with users. The directive chatbot was named D-Bot and the non-directive chatbot N-Bot. Both chatbots were structured around the four stages of the GROW coaching model: Goal, Reality, Options, and Will (Whitmore, 2017), which provided a consistent process framework across both conditions, ensuring that any observed differences in participant responses could be attributed to coaching style rather than structural variation in the session. Table 1 is an overview of the prompt structure and Figure 2 provides an example of the look and feel of the chatbot interface. Apart from the coaching-style instruction section, all remaining prompt structures, GROW process stages, user interface elements, and platform settings were kept consistent across both chatbot conditions to minimise the influence of unrelated LLM response variability.

**Table 1:** Chatbot prompt structure.

Section	Prompt summary
Description	Provided a summary of the purpose of the chatbot and its relation to the research being conducted.
Systems instructions	Outlined the AI chatbot’s coaching role, context, behaviour and style, referencing the Heron model to explain directive and non-directive elements. Defined the coaching scope, limited discussion of therapeutic issues, and set rules requiring participants to complete each session fully. This was the only section where there was a significant difference between the directive and the non-directive AI chatbots. It represented 8% of the prompt word count.
Onboarding instructions	Welcomed the coachee, introduced the researcher and the research purpose, named the AI chatbot, and aimed to build rapport with the coachee.
Greeting	Aimed to build rapport with the coachee through examples of conversational greetings and opening questions about their goals for the session.
Goal	Covered how to help the coachee set a clear, actionable goal at the start of the GROW coaching process.
Reality	The coachee was asked several questions to clarify their current reality on the topic discussed in the goal section.
Options	The coachee was asked to consider options to help achieve their goal.
Will (actions)	The coachee specify and commit to their planned actions for achieving their goals.
Closing	The final step involved expressing gratitude to the coachee for their participation in the session and sharing the link to the next section of the survey, enabling them to complete the research process.

Note the difference in level of directiveness in the last message in both conversations in Figure 2 with D-bot (left-hand side) clearly suggesting a course of action with implications,. while N-Bot (right-hand side) providing no direct suggestions but instead providing a summary and then asking a question.

The theoretical basis for differentiating the two chatbot styles was Heron (2001) six-category intervention analysis model, operationalised using behavioural statements from De Haan and Nilsson (2017) Coaching Behaviour Questionnaire (CBQ). These conversational statemens where used as examples in the LLM prompts design for D-Bot and N-Bot, respectively, to help ensure adherence to the theory of directiveness (see Table 2).

**Table 2:** Directive and non-directive statements used to design the chatbots (De Haan and Nilsson, 2017).

Coaching approach	Statements
Directive approach	Let them know how a task, meeting, or job can be done really well.
	Tell them how to get started on a task.
	Say: “I would suggest you do….”
	Make suggestions regarding “homework” for the next meeting.
	Advise them of what action to take.
	Advocate a particular solution or approach.
	Show them how to correct their mistakes.
	Indicate it might be a good idea to change.
	Persuade them to take a particular approach.
	Give them an example of how I would approach the issue.
	Help them to state their present understanding of the issues.
	Recommend how to approach or do something.
	Propose what I believe to be the best course of action.
Non-directive approach	Ask them how they feel about a current difficulty.
	Let them get on with finding their own answers.
	Ask for their interpretation of a particular situation.
	Say: “What else would you like to cover?.”
	Point out that they could look at the issue in a different way.
	Ask for desired outcomes for the meeting.
	Ask them what next to explore.
	Help them to reflect on their experiences.
	Ask them how they can apply what they have learnt.
	Ask open questions to promote new insights.
	Encourage them to find their own solutions and answers.
	Inquire into what they want to achieve.

D-Bot was positioned on Heron’s prescriptive-catalytic gradient between commanding prescription and consultative prescription, employing directive interventions such as providing suggestions, recommending courses of action, giving examples, and advising on next steps. N-Bot was positioned at the facilitative self-direction end of the gradient, employing non-directive interventions such as asking open questions, encouraging self-reflection, inviting coachees to identify their own solutions, and exploring desired outcomes. This design aimed to ensure that the two chatbots represented meaningfully distinct and theoretically grounded implementations of directive and non-directive coaching styles, respectively. Before launching the research study, the two chatbots were tested and validated by eight experienced business coaches. Four of the coaches have master’s degrees in coaching, while three others have studied at master’s level. All eight coaches responded in the affirmative, indicating that the difference in directive character between the two chatbots was clear. The following are some verbatim quotes:

*They have very different feels. The non-directive[N-Bot] has a much softer personal approach at the start. I found the directive model [D-Bot] quite impersonal to begin with.*

*Both [chatbots] may serve a different purpose. When I want ready answers, go to directive and [when] I want soul searching then non-directive.*

The chatbot validation test showed that the two AI chatbots had clearly different styles, which the experienced coaches identified as directive and non-directive.

### Participants

Participants were recruited through Prolific, a crowdsourcing platform widely used in social science and AI-related research (Palan and Schitter, 2018; Terblanche, 2024b). Pre-screening filters were applied to ensure the sample represented professional working millennials based in the United Kingdom. Inclusion criteria required participants to be aged between 25 and 45 years (millennial age), employed full-time, English first-language speakers, and holders of a post-secondary qualification. The age filter was split equally between 25 and 35 years (50%) and 36–45 years (50%), and the qualification filter targeted undergraduate (60%), masters (40%), and doctoral (10%) level respondents. The United Kingdom was selected as the recruitment location given the substantial Prolific user base in that country, English as the primary language, and the practical advantage of a single time zone enabling both study stages to be administered at comparable times. Participants were remunerated at the rate recommended by Prolific. Invitations were distributed to approximately 6,500 eligible respondents, with each study stage concluding once 80 valid responses had been recorded.

A between-subjects design was employed, with participants in stage one (D-Bot) explicitly excluded from participation in stage two (N-Bot) using Prolific’s built-in exclusion functionality. This ensured independence between the two sample groups and prevented carry-over effects.

### Measurement framework and survey instrument

To assess millennial ratings across the two chatbot conditions (directive vs. non-directive), this study employed four validated measurement instruments. Technology adoption was measured using selected constructs from the AI Device Use Acceptance (AIDUA) framework (Gursoy et al., 2019), which draws on the Unified Theory of Acceptance and Use of Technology (Venkatesh et al., 2003; Venkatesh et al., 2012). The AIDUA and UTAUT frameworks have demonstrated effectiveness in measuring adoption attitudes toward AI chatbots across service and coaching contexts (Lin et al., 2020; Terblanche and Kidd, 2022) and was selected for its specific applicability to AI-enabled interactions. Social influence was excluded from the instrument as the anonymous online recruitment mechanism used in this study precluded meaningful measurement of peer or managerial influence.

Goal attainment was assessed using Goal Theory, which is anchored by five core principles guiding effective goal setting: objectives should be precise and explicit; goals must strike a balance between being sufficiently challenging and realistically achievable; commitment should be established and sustained; consistent feedback on progress is essential; and goal complexity should remain demanding yet attainable (Locke and Latham, 2002). Goal setting and achievement underpins the well-established GROW coaching model (Whitmore, 2017) which is particularly well-suited to chatbot-enabled coaching given its structured, sequential nature (Grant, 2006; Terblanche, 2024b). Working alliance was measured using the Working Alliance Inventory (WAI) (Horvath and Greenberg, 1989), which assesses three dimensions of the coach-coachee relationship: bond (trust, respect and confidence), goal (collaborative agreement on desired outcomes), and task (agreement on the activities needed to achieve those goals). The WAI has been applied previously in AI coaching research, with Terblanche (2024b) finding that coachees experienced AI chatbots as psychologically safe and non-judgmental partners in the coaching process. Grant (2014a) found that goal and task dimensions were twice as impactful as the bond dimension in predicting successful coaching outcomes, a finding with particular relevance for AI coaching where bond formation may differ from human-to-human coaching relationships.

Finally, personality traits were measured using the Big Five instrument (Costa and McCrae, 2008), which assesses extraversion (the way a person engages in social interactions), agreeableness (describes those who trust others, avoid conflict and are kind and cooperative), conscientiousness (individuals who are rational, informed and competent), emotional stability (reflecting the level of negative emotions and thoughts experienced), and openness to experience (receptiveness to new ideas and experiences). The instrument was selected on the basis of evidence linking personality traits, particularly extraversion, to perceived coaching effectiveness (Jones et al., 2014) and has been previously applied in AI coaching research to examine the relationship between coachee personality and chatbot adoption (Terblanche and Heyns, 2020).

The survey instrument was deployed in two sequential parts using an online survey platform and comprised 74 items rated on a five-point Likert scale (1 = strongly disagree, 5 = strongly agree). Part one was completed before the chatbot session and part two immediately after. See Appendix A for the complete survey.

Personality traits were assessed using the mini-IPIP scale (Donnellan et al., 2006), a validated 20-item short form of the Goldberg Big Five instrument (Goldberg, 1992), measuring extraversion, agreeableness, conscientiousness, emotional stability, and openness to experience (four items per construct).

Technology adoption was measured using the AI Device Use Acceptance (AIDUA) framework (Gursoy et al., 2019), adapted to reference AI coaching chatbots in line with modifications used by (Terblanche, 2024b). The instrument assessed eight constructs: performance expectancy, effort expectancy, emotion, hedonic motivation, anthropomorphism, attitude toward the chatbot, willingness to accept AI, and objection to AI use. Social influence was excluded as the anonymous online recruitment mechanism precluded meaningful measurement of peer or managerial influence.

Goal attainment was measured using a six-item instrument drawn from Terblanche (2024b), grounded in goal theory (Grant, 2006; Locke and Latham, 2002) and previously validated in an AI coaching chatbot context. Working alliance was assessed using a 12-item adaptation of the Working Alliance Inventory (WAI) (Horvath and Greenberg, 1989), modified to reflect interaction with an AI coaching chatbot rather than a human coach. The WAI assessed three dimensions: bond (trust, respect and confidence between coachee and chatbot), goal (collaborative agreement on desired outcomes), and task (agreement on the activities required to achieve those goals).

### Data collection procedure

Data collection proceeded in two stages. Stage one launched on the Prolific platform on 13 August 2025, directing participants to D-Bot. In step one (pre-survey), participants entered their anonymous Prolific ID, completed the demographic and Big Five sections, and received the D-Bot link. In step two, they completed a 5–15 min coaching session on the CoachBot.AI platform. Participants who successfully engaged and completed their coaching conversation (i.e., worked progressively through the four GROW steps) with the chatbot, received the link to the second survey. The bot was specifically designed to check for completion of the conversation. Data from participants who did not complete the entire coaching conversation with the respective chatbot were excluded from the study as they were unable to progress to the second survey. In step three, successful participants completed the AIDUA, goal attainment, and working alliance sections of the survey and received a completion code for Prolific remuneration. Stage two followed an identical procedure on 14 August 2025 using N-Bot, with all stage one participants excluded.

### Data analysis

Two-way mixed ANOVA (St and Wold, 1989) was used to determine whether statistically significant differences existed between the directive and non-directive chatbot groups across technology adoption, working alliance, and goal attainment. Internal consistency of the survey constructs was assessed using Cronbach’s alpha (Keppel, 1991). Separate ANCOVA (Rutherford, 2011) models were conducted to examine interaction effects between chatbot condition and Big Five personality traits on technology adoption, working alliance, and goal attainment outcomes. Personality traits were treated as continuous moderator variables. Moderation was assessed through the interaction between chatbot condition (directive vs. non-directive) and personality trait scores. Effect sizes were reported using Cohen’s d to quantify the magnitude of observed differences between groups (Lakens, 2013). Statistical significance was evaluated using *p*-values, with a threshold of *p <* 0.05. All statistical analysis was conducted independently by a statistician.

### Ethical considerations

Ethical approval was obtained from the researchers’ institution (project no. 33609) prior to data collection. Informed consent was obtained from all participants before they commenced the study. Participation was voluntary and anonymous, with data used exclusively for academic research purposes. The CoachBot.AI platform is GDPR-compliant, and all data was stored and managed in accordance with institutional data governance requirements.

## Results

### Sample characteristics

Both chatbot stages yielded 79 fully completed surveys (*n =* 79 D-Bot; *n =* 79 N-Bot). The sample size is within the range of prior experimental AI coaching chatbot studies in this emerging field. For example, Terblanche (2024a) employed samples of *n =* 126 and *n =* 116, respectively, in a comparative chatbot experiment using similar outcome measures. While the present study included a somewhat smaller sample, it remained appropriate for the exploratory nature of the research and was sufficient to detect statistically significant differences across several key constructs. Gender distribution was comparable across both groups. Age distribution was broadly similar, though the N-Bot sample showed a slight bias toward the 36–40 year age group. Both samples were weighted toward junior and middle management job levels, with high levels of educational attainment consistent with the Prolific recruitment criteria requiring post-secondary qualifications. General AI usage was high across both groups, with 89% of D-Bot respondents and 90% of N-Bot respondents reporting use of general AI tools such as ChatGPT or Gemini at least once per week, confirming the tech-savvy profile of the millennial sample.

### Chatbot interaction analysis

Analysis of session interaction data recorded by the chatbot platform revealed meaningful differences in engagement patterns between the two chatbot conditions. D-Bot produced a mean message count of 23.8, while N-Bot produced a mean of 29.7, with comparable ranges across both conditions (D-Bot: 11–57; N-Bot: 17–61). The higher message count for N-Bot reflects the exploratory, question-driven nature of non-directive coaching, which requires more conversational exchanges to navigate the GROW model stages compared to the more direct guidance offered by D-Bot.

### Data reliability

Internal consistency was assessed using Cronbach’s alpha across all 17 constructs. Values ranged from 0.75 to 0.95, all exceeding the accepted threshold of 0.70 (Keppel, 1991), confirming strong reliability across the full survey instrument. Construct-level alpha values are reported in Table 3.

**Table 3:** Summary results of chatbot main effect for comparing D-Bot versus N-Bot.

Questionnaires	Construct	Abbreviation	Conbach’s Alpha	Cohen’s d	D-Bot (*n =* 79)		N-Bot (*n =* 79)		*p*-value
					Mean	SD	Mean	SD	
Big 5 personality types	Extraversion	EXT	0.87						
	Agreeableness	AGR	0.83						
	Conscientious	CON	0.75						
	Neuroticism	EMO	0.75						
	Openness	OPE	0.81						
AI device user acceptance (AIDUA)	Performance expectancy	PE	0.91	0.38***	3.83	0.74	3.53	0.83	0.02*
	Effort expectancy	EE	0.86	0.19	4.26**	0.51	4.15**	0.66	0.24
	Emotion	EM	0.94	0.17	3.87**	0.82	3.73	0.86	0.27
	Hedonic motivation	HM	0.90	0.09	3.61	0.82	3.68	0.79	0.59
	Anthropomorphism	AN	0.89	0.06	1.98	0.94	2.03	0.86	0.71
	Attitude towards AI coaching chatbot	AT	0.85	0.25	3.91**	0.74	3.73	0.72	0.12
	Willingness to accept AI	WA	0.90	0.08	3.82**	0.79	3.76	0.89	0.64
	Objection to AI use	OU	0.75	0.12	3.22	0.8	3.31	0.8	0.46
Goal attainment	Goal attainment	GA	0.95	0.28***	3.8	0.84	3.57	0.79	0.08*
Working alliance (WA)	Task	TK	0.89	0.31***	3.84	0.78	3.59	0.79	0.05*
	Bond	BD	0.88	0.13	3.07	0.9	2.96	0.86	0.43
	Goal	GL	0.85	0.59***	4.03	0.57	3.63	0.76	0.01*

*Indicates a significant *p*-value. **Indicates a high mean. ***Indicates potentially meaningful Cohen’s d.

### ANOVA results: directive versus non-directive chatbot ratings

A two-way mixed ANOVA was conducted to assess differences between D-Bot and N-Bot across technology adoption, goal attainment, and working alliance constructs. Results are summarised in Table 3.

Regarding technology adoption, a statistically significant difference was found in favour of D-Bot for performance expectancy (PE; *p* = 0.02), with a practically meaningful effect size (Cohen’s d = 0.38). No significant differences were observed across the remaining six AIDUA constructs. For goal attainment, D-Bot was rated higher than N-Bot at a near-significant level (GA; *p* = 0.08; Cohen’s d = 0.28), suggesting a trend in favour of the directive chatbot. For working alliance, statistically significant differences in favour of D-Bot were found for both the task construct (TK; *p* = 0.05; Cohen’s d = 0.31) and the goal construct (GL; *p* = 0.01; Cohen’s d = 0.59). No significant difference was found for the bond construct.

### ANCOVA results: personality traits and chatbot ratings

ANCOVA was employed to examine interaction-based moderation effects between chatbot condition and Big Five personality traits across technology adoption, working alliance, and goal attainment constructs. Least-squares means graphs were used to visualise adjusted group means after controlling for covariate effects.

Statistically significant interactions were identified for three personality traits, namely extraversion (EXT), conscientiousness (CON), and openness to experience (OPE), across the performance expectancy and working alliance constructs. No significant interactions were found for agreeableness or emotional stability.

**Figure 3:**
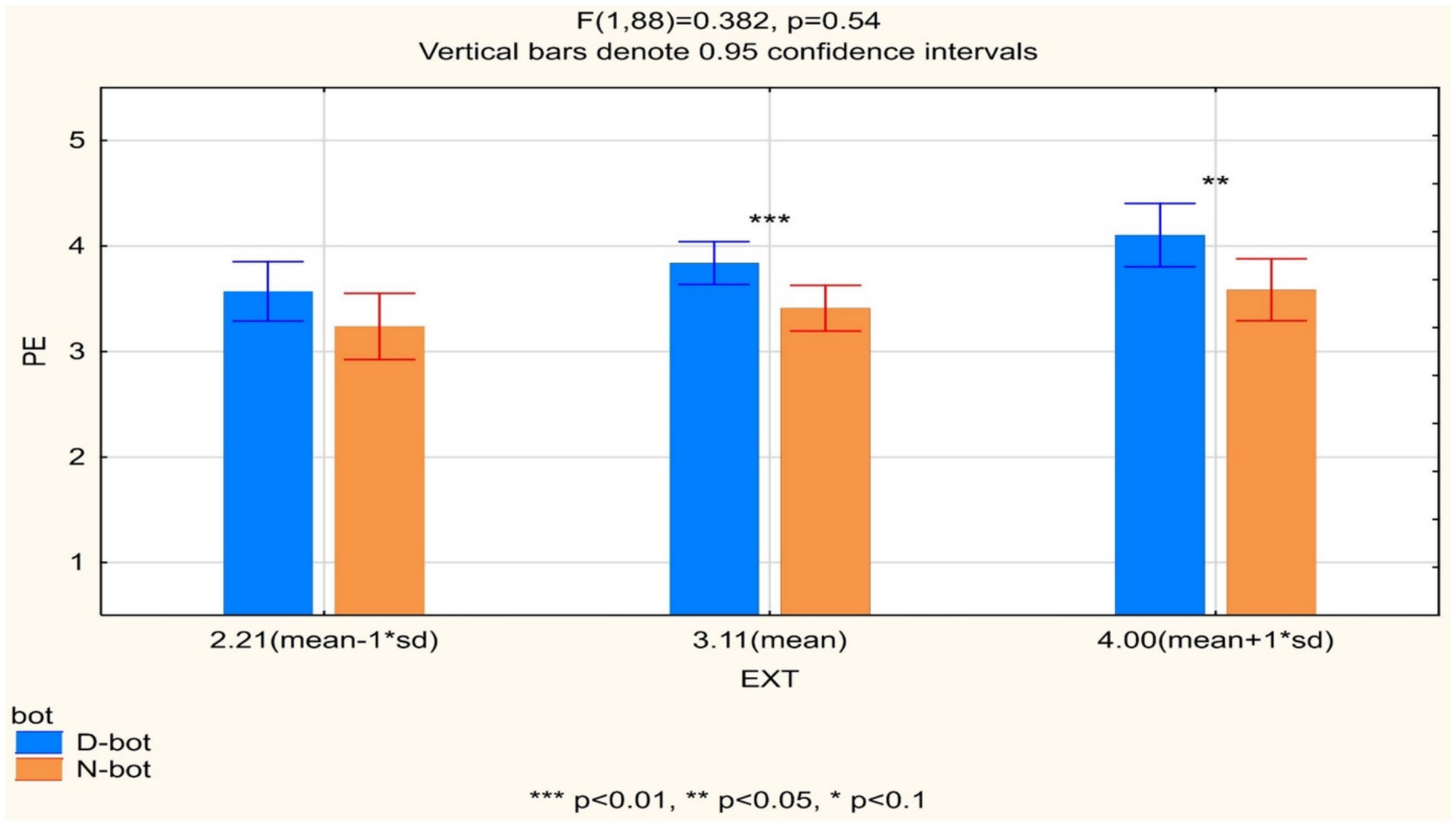
LS-means graph: performance expectation and extraversion.

For performance expectancy, significantly higher scores for D-Bot was observed as extraversion scores increased (*p <* 0.05) as indicated in Figure 3 above, and as conscientiousness scores increased (*p <* 0.01), indicating that respondents who are more socially engaged and self-disciplined show higher scores in favour of the directive chatbot’s performance-oriented approach.

A significant interaction effect was observed between chatbot condition and both extraversion (*p <* 0.05) and conscientiousness (*p <* 0.05) for the working alliance task construct. The directive chatbot was rated more favourably on task alignment among respondents with higher levels of extraversion and conscientiousness, mirroring the pattern observed for performance expectancy and suggesting that structured, goal-directed coaching interactions may resonate more strongly with these personality profiles.

For the working alliance goal construct, two significant interaction effects emerged. First, an interaction between chatbot condition and extraversion (*p <* 0.01) indicated that respondents with lower levels of extraversion rated the directive chatbot more favourably for goal alignment. Second, an interaction between chatbot condition and openness to experience (*p <* 0.05) indicated that respondents higher in openness rated the directive chatbot more favourably on the goal construct, suggesting that individuals characterised by curiosity and receptiveness to new ideas may respond positively to directive, goal-focused AI coaching interactions.

These findings are summarised in Table 4. Collectively, the ANCOVA results suggest that extraversion, conscientiousness, and openness to experience may moderate millennial responses to directive AI chatbot coaching.

**Table 4:** Inferential statistics summary.

Dependant variable	Trend	Personality construct	*p*-value
Performance expectation	Increases toward D-Bot	As extraversion increases	*p <* 0.05
Performance expectation	Increases toward D-Bot	As conscientiousness increases	*p <* 0.01
Working alliance task	Increases toward D-Bot	As extraversion increases	*p <* 0.05
Working alliance task	Increases toward D-Bot	As contentiousness increases	*p <* 0.05
Working alliance goal	Increases toward D-Bot	As extraversion decreases	*p <* 0.01
Working alliance goal	Increases toward D-Bot	As openness increases	*p <* 0.05

## Discussion

This study asked four research questions in relation to millennial users: (1) What are the differences in technology adoption between a directive vs. non-directive AI coaching chatbot? (2) What are the differences in working alliance between a directive vs. non-directive AI coaching chatbot? (3) What are the differences in goal attainment between a directive vs. non-directive AI coaching chatbot? (4) What is the role of personality traits of millennials in their technology adaption. Working alliance and goal attainment when using a directive vs. non-directive AI coaching chatbot?

The results favour the directive chatbot across one (but important) construct of technology adoption, two of the three working alliance constructs, and a tendency for goal attainment. There are also personality-based moderating effects. These results challenge the prevailing non-directive paradigm in professional coaching and carry implications for AI coaching design, coaching practice, and organisational learning and development.

Before discussing the results in terms of each research question, it is worth noting that the two chatbots were independently validated by eight experienced business coaches prior to the study, all of whom confirmed that the directive and non-directive characters of D-Bot and N-Bot were clearly distinguishable. Interestingly, the coaches themselves expressed a preference for the non-directive chatbot, a finding consistent with De Haan and Nilsson (2017) observation that coaches over 40 tend to self-report as less directive than their younger counterparts. This contrast between coach preference and coachee ratings of the chatbots is itself noteworthy and reinforces the relevance of the study’s central finding.

### Technology adoption and performance expectancy

For the first research question, the ANOVA results identified performance expectancy as the only technology adoption construct showing a statistically significant difference between the two chatbot conditions. Performance expectancy has been shown in previous studies as the strongest predictor of AI chatbot adoption across service, educational, and coaching contexts (Kuberkar and Singhal, 2020; Terblanche and Kidd, 2022; Van der Merwe and Terblanche, 2025). Therefore, even though it is the only technology adoption construct that shows a difference, it can be considered an important one (Terblanche and Kidd, 2022).

Cognitive load theory offers a possible explanation for this result. Sweller (2011) proposed that problem-solvers often possess a goal but lack the know-how of to reach it, generating high intrinsic cognitive load. The directive chatbot most likely reduced this load by providing information and structured options, reducing the cognitive load (Atkinson et al., 2000). The high effort expectancy scores across both conditions (D-Bot: 4.26; N-Bot: 4.15) suggests that both chatbots were equally accessible and easy to use, therefore pointing coaching style (directive vs. non-directive) rather than usability as the driver of the performance expectancy advantage. Additionally, algorithmic appreciation, defined as the tendency for individuals to follow advice more readily when they believe it originates from an algorithm (Logg et al., 2019), and the psychological safety of AI interactions (Terblanche et al., 2024) may have further elevated performance expectancy for the directive condition.

### Working alliance

For the second research question, users rated D-Bot higher for goal and task constructs of working alliance, with no significant difference in the bond construct. This pattern aligns with Grant (2014a) finding that goal and task dimensions of working alliance are twice as predictive of coaching success as the bond dimension, a distinction particularly relevant in AI coaching contexts where affective bond formation may inherently differ from human-to-human coaching relationships. The findings indicate that millennials experienced the directive chatbot as a more effective collaborative partner for establishing clear goals and agreeing on concrete action steps, which are the dimensions most consequential for coaching outcomes.

This result affirms Terblanche (2021) finding that coachees undergoing transitions valued directive input in the form of frameworks and informed challenge and aligns with the Heron model’s informing and prescribing interventions, which position the coach as a provider of knowledge and structured guidance (Heron, 2001). The implication is that for AI coaching, structure and clarity in goal and task alignment appear to matter more to coachees than the warmth or relational quality of the interaction, or bond construct.

### Goal attainment

For the third research question the directive chatbot was rated higher for goal attainment indicating a trend. While this does not meet the conventional *p <* 0.05 threshold, the consistent directional trend and practically meaningful effect size suggest that the result carries practical significance, particularly given the importance of goal attainment as a central coaching outcome (Grant, 2006; Locke and Latham, 2002). The directive chatbot’s suggestions of ideas, options, and structured steps appears to have facilitated greater clarity in goal formulation and stronger motivation for goal pursuit, consistent with goal theory’s proposition that clear, challenging, and manageable goals with regular feedback lead to higher achievement (Locke and Latham, 2002). These findings offer support for Ives (2008) assertion that a directive coaching approach is appropriate when goal attainment support is the primary objective.

### Personality traits

For the final research question, the ANCOVA results revealed that extraversion, conscientiousness, and openness to experience each significantly affected the scoring differences between the two chatbot conditions. Highly conscientious respondents, characterised by goal orientation, self-discipline, and preference for structured approaches (Bates et al., 2023) showed a higher score for D-Bot across both performance expectancy and working alliance task. This is consistent with Barnett et al. (2015) finding that conscientiousness positively predicts engagement with structured learning technologies, and with Bates et al. (2023) observation that conscientiousness correlates strongly with learning goal orientation.

Higher extraversion was associated with significantly greater scores for D-Bot on performance expectancy and working alliance task, but a paradoxical decrease in scoring for D-Bot on working alliance goal as extraversion increased. This apparent contradiction may reflect the finding by Barnett et al. (2015) that extraversion can have an unexpectedly negative effect on actual technology use due to the extraverted prefernces for external stimulation and social interaction. Applied to goal alignment, highly extraverted coachees may require richer, more interactive conversational dynamics to collaboratively construct their goals. This suggests even directive chatbots may need augmented social features or even human coaches to fully meet the needs of highly extraverted users.

Respondents high in openness to experience scored D-Bot higher on working alliance goal. Given the strong link between openness and learning goal orientation (Bates et al., 2023), the directive chatbot’s provision of information and suggetions appears to have aligned well with the curious, intellectually receptive profile of high-openness users, enhancing the perceived value of the goal-setting process.

The personality trait findings suggest that a one-size-fits-all approach to AI coaching chatbot design is suboptimal. This supports previous findings and suggestions of adaptive AI coaching systems capable of dynamically adjusting coaching modality, style and approach based on user personality profiles (Terblanche et al., 2024). This result is also echoed in human coaching through De Haan and Nilsson (2017) assertion that the most effective coaches are those who adaptively deploy the full range of coaching styles at the appropriate moment.

### Theoretical contributions

This exploratory study offers four tentative theoretical contributions to coaching and human–AI interaction research.

Firstly, it challenges the dominance of the non-directive paradigm in coaching theory by showing that ratings are different in AI contexts. Contrary to established assumptions (Updated ICF Core Competencies, 2019), a directive style was favoured to some extent, indicating that coaching effectiveness could be dependent on interaction context (human vs. AI). This introduces the idea that the effectiveness of coaching approaches could depends on the context in which they are applied.

Secondly, the findings suggest possible extensions to working alliance theory by demonstrating that in AI coaching, task and goal dimensions are important, while bond is less so. This suggests that the structure of the working alliance could be different in human–AI interactions, with transactional clarity seemingly more relevant than relational depth as the primary mechanism of effectiveness.

The third contribution is that the findings suggest that coaching style may warrant consideration as a potential antecedent of performance expectancy. This suggests that interaction design, particularly directive guidance could play an important role in shaping how useful AI systems are perceived to be.

Finally, the study contributes to generative AI coaching theory by suggesting that personality traits may moderate coaching style rating, establishing personality as a condition for AI coaching effectiveness.

These contributions position directive versus non-directive coaching as a context- and user-dependent design variable, extending coaching theory into the world of AI coaching.

### Implications for practice

These findings carry practical implications for multiple stakeholders. For professional coaches, the results reinforce the case made by Grant (2014b), Wood (2015), Terblanche (2021) and Cavanagh (2006) that directive interventions, namely suggesting ideas, sharing frameworks, and providing expert input, are legitimate and valued coaching behaviours that should be part of every coach’s repertoire. The findings also suggest that directive AI chatbots could potentially serve effectively as pre-session or between-session support tools, helping coachees clarify goals and reduce cognitive load before human coaching sessions, thereby extending the reach and reducing the cost of professional coaching.

For organisational learning directive AI chatbots may represent a cost-effective mechanism for delivering structured, goal-based developmental coaching to junior leaders and early-career employees who would typically be excluded from formal coaching programmes on cost grounds; and who require guidance and mentoring in addition to non-directive coaching. Organisations may further optimise outcomes by integrating personality assessments such as the Big Five to match employees to chatbot coaching styles aligned with their traits.

For AI coaching platform developers, the study demonstrates that coaching style is not a neutral design decision but an important feature that directly affects user acceptance and coaching outcomes. Developers should incorporate directive elements like structured prompts, examples, and guidance into their systems, while building personality-adaptive features that allow the degree of directiveness to be calibrated to individual user profiles. Attention to the needs of highly extraverted users, who may require richer social and collaborative features to achieve satisfactory goal alignment, is a specific design priority identified by this research.

Finally, for coaching bodies such as the International Coaching Federation and the European Mentoring and Coaching Council, these findings suggest that the behavioural guidelines governing directive versus non-directive coaching practice warrant reconsideration. The evidence presented here, alongside the findings of Stober and Grant (2010), Terblanche (2021), and Wood (2015), indicates that the directive vs. non-directive distinction is a matter of context rather than dogma. Coaching standards should better define the contexts in which directive interventions are appropriate and move away from a framing that positions directiveness as inherently contrary to good coaching practice. Furthermore, coaching bodies who propose AI coaching standards must take note of the preference for a directive approach and adjust their AI coaching recommendations in line with the findings of this study.

### Limitations and future research

Several limitations of this study should be acknowledged. The sample comprised UK-based, English first-language, graduate-level millennials recruited via Prolific. While this enhanced internal consistency and ensured a high-quality respondent pool, the findings should not be generalised to non-graduate populations, multilingual settings, or regions with lower digital literacy. Furthermore, all interactions occurred on a single platform with a common user interface, with only targeted prompt differences distinguishing D-Bot from N-Bot. The web-based design and shared user experience may have influenced performance expectancy, goal attainment, and working alliance outcomes, potentially limiting transferability to other chatbot architectures such as mobile delivery via WhatsApp or alternative modalities such as voice or avatar-based coaching.

Another limitation is that respondents engaged in a single session of approximately 5–15 min. Such brief exposure, combined with the possible novelty of using a coaching chatbot for the first time, may have influenced performance expectancy and working alliance scores. A longitudinal design would be required to measure whether preferences and outcomes change as coachees gain familiarity with AI coaching over multiple sessions.

Given the number of dependent variables examined, the study carries a risk of Type I error inflation. The findings should therefore be interpreted as exploratory and theory-building rather than confirmatory. Future studies should use larger samples.

In terms of future research, the study should be replicated with broader and more diverse populations, including non-UK respondents and Generation Z participants, who according to Deloitte (2024) demonstrate an even stronger propensity for AI chatbot adoption than millennials. Testing whether the directive preference finding holds across different generational cohorts and cultural contexts would strengthen the generalisability of these results.

The present study raises questions about human coaching. A study replicating this design with a sample of professional coaches would be valuable. De Haan and Nilsson (2017) found that coachees consistently rated their coaches as more directive than coaches rated themselves, and the anecdotal preference for N-Bot expressed by the validation coaches in this study adds further weight to the suggestion that coaches and coachees may hold systematically different preferences. No study has yet examined this divergence using a consistent methodology across both populations.

Future research should also investigate adaptive AI coaching systems that dynamically adjust coaching style based on in-session needs and user personality profiles. Shumanov and Johnson (2021) demonstrated that aligning chatbot personality with user personality increases engagement. Finally, research examining human-AI hybrid coaching models, combining human coaching sessions with regular AI chatbot interactions would help determine whether such models produce superior goal attainment and working alliance outcomes compared to human-only coaching interventions, and how directive AI tools might best complement rather than replace the human coaching relationship.

## Conclusion

This study set out to determine whether tech-savvy millennials, the dominant generational cohort in the global workforce, prefer a directive or non-directive coaching style when engaging with a Gen AI coaching chatbot. Measuring ratings across technology adoption, working alliance, goal attainment, and personality traits, this study identified tentative evidence in favour of the directive coaching style. This higher rating was most pronounced for performance expectancy, the dominant predictor of AI technology adoption and for the goal and task dimensions of working alliance, which are the dimensions most predictive of coaching success. Goal attainment showed a tendency for the directive chatbot, and personality analysis revealed that extraversion, conscientiousness, and openness to experience each moderated coaching style preference.

These findings raise questions about the prevailing assumption that non-directive coaching is inherently superior, albeit in a very specific context. They suggest that for millennials engaging in goal-directed AI coaching, structure, clarity, and the provision of ideas and frameworks enhance perceived usefulness and coaching effectiveness, at least in the context of the present study. They also demonstrate that coaching style preference is not uniform but most likely shaped by individual personality, pointing toward the development of adaptive AI coaching systems capable of calibrating their approach to the user.

This research contributes to the evolving research on AI coaching by providing the first empirical exploration of directive versus non-directive style preferences in AI coaching chatbots. These insights have implications for the design and deployment of AI coaching platforms, for organisational learning and development practice, for professional coaches navigating the integration of AI into their work, and for coaching bodies reconsidering the contextual boundaries of their behavioural guidelines. As AI coaching continues to develop, understanding what coachees actually prefer, rather than what conventions prescribe, will be essential to realising the full potential of AI as a coaching tool.

## Data Availability

The raw data supporting the conclusions of this article will be made available by the authors, without undue reservation.
