# Supplementary material for: Rethinking directiveness in AI coaching chatbots
Source: Front Psychol. 2026 Jun 23;17:1822088. doi: 10.3389/fpsyg.2026.1822088 (PMC13337845; doi:10.3389/fpsyg.2026.1822088)
Supplement: Supplementary file 1 [file Supplementary_file_1.docx]

# Appendix A – Survey instrument

*Table 5: Demographic questions*

| Response category | Response options |
| --- | --- |
| Age | 25 to 30 years  31 to 35 years  36 to 40 years  41 to 45 years |
| Gender | Male  Female  Other |
| Current job level | Entry-level/Junior  Intermediate/Experienced professional  Senior Professional/Specialist  Team Leader/Supervisor  Manager  Senior Manager  Director/Head of department  Executive (e.g. CEO, CFO, COO)  Business Owner/Entrepreneur |
| Educational level | High school or less  Undergraduate degree  Honours degree  Master’s degree  Doctoral degree  Other |
| How often do you use generative AI tools, such as ChatGPT (or similar tools, such as Claude, Gemini, or Copilot)? | Daily  A few times a week  Once a week  Rarely (less than once a month)  Never |

*Table 6: Big Five questionnaire*

| Item | Factor | Text |
| --- | --- | --- |
| 1 | EXT | Am the life of the party |
| 2 | AGR | Sympathise with others' feelings |
| 3 | CON | Get chores done right away |
| 4 | EMO | Have frequent mood swings |
| 5 | OPE | Have a vivid imagination |
| 6 | EXT | Don't talk a lot (R) |
| 7 | AGR | Am not interested in other people's problems (R) |
| 8 | CON | Often forget to put things back in their proper place (R) |
| 9 | EMO | Am relaxed most of the time (R) |
| 10 | OPE | Am not interested in abstract ideas (R) |
| 11 | EXT | Talk to a lot of different people at parties |
| 12 | AGR | Feel others' emotions |
| 13 | CON | Like order |
| 14 | EMO | Get upset easily |
| 15 | OPE | Have difficulty understanding abstract ideas (R) |
| 16 | EXT | Keep in the background (R) |
| 17 | AGR | Am not really interested in others (R) |
| 18 | CON | Make a mess of things (R) |
| 19 | EMO | Seldom feel blue (R) |
| 20 | OPE | Do not have a good imagination (R) |

(R) – Reverse scored item

A 5-point Likert scale was used to measure agreement with the statements.

*Table 7: AIDUA statements*

| Construct | Survey statements |
| --- | --- |
| **Performance**  **expectancy (PE)** | Using the coaching chatbot helped me to achieve my goals more quickly. |
|  | Using the coaching chatbot enhanced my effectiveness to achieve my goals. |
|  | Using the coaching chatbot enhanced my motivation to achieve my goals. |
|  | I found the coaching chatbot useful for improving my skills to achieve my aims. |
|  | I found the coaching chatbot useful for my future coaching needs. |
| **Effort expectancy (EE)** | Overall, I believe the coaching chatbot was easy to use. |
|  | Using the AI chatbot for coaching took too much time. (R) |
|  | I found it easy to communicate with the coaching chatbot. |
|  | Learning to use the chatbot for coaching was easy for me. |
|  | My coaching activities with the chatbot were clear and understandable. |
|  | I found it easy to achieve my aims with the coaching chatbot. |
| **Emotion (EM)** | I felt relaxed after using an AI coaching chatbot. |
|  | I felt content after using an AI coaching chatbot. |
|  | I felt hopeful after using an AI coaching chatbot. |
|  | I felt satisfied after using an AI coaching chatbot. |
|  | I felt pleased after using an AI coaching chatbot. |
| **Hedonic motivation (HM)** | I think using the coaching chatbot was fun. |
|  | I think using the coaching chatbot was entertaining. |
|  | I think using the coaching chatbot was enjoyable. |
|  | I think that interacting with AI devices is fun. |
| **Anthropomorphism (AN)** | AI coaching chatbots have a mind of their own. |
|  | AI coaching chatbots have consciousness. |
|  | AI coaching chatbots have their own free will. |
|  | AI coaching chatbots will experience emotions. |
| **Attitude towards AI coaching chatbot (AT)** | Using a chatbot for coaching is a good idea. |
|  | I found that the actual process of engaging with the coaching chatbot was pleasant. |
|  | I would like to use the chatbot to achieve my aims. |
|  | The chatbot made working towards my aims more interesting. |
|  | Using the chatbot was frustrating for me. (R) |
| **Willingness to accept AI (WA**) | I am willing to receive AI coaching services. |
|  | I feel happy to interact with AI coaching chatbots. |
|  | I am likely to interact with AI coaching chatbots in the future. |
| **Objection to AI use (OU)** | I think that information is processed in a less humanised manner by the AI coaching chatbot. |
|  | I prefer human contact in coaching. |
|  | People need emotion exchanged during coaching sessions. |
|  | Interaction with AI coaching chatbots lacks social contact. |

(R) – Reverse scored item

A 5-point Likert scale was used to measure agreement with the statements.

*Table 8: Goal attainment statements*

| Construct | Survey statements |
| --- | --- |
| **Goal attainment (GA)** | Using an AI coaching chatbot would help me to achieve my goals more quickly. |
|  | Using an AI coaching chatbot would enhance my effectiveness to achieve my goals. |
|  | Using an AI coaching chatbot enhances my motivation to achieve my goals. |
|  | I find the AI coaching chatbot useful for improving my skills to achieve my goals. |
|  | I find the AI coaching chatbot useful for equipping me to achieve my goals. |
|  | I would find the AI coaching chatbot useful for my future coaching needs. |

A 5-point Likert scale was used to measure agreement with the statements.

*Table 9: Working Alliance Inventory (WAI) statements*

| Construct | Survey statement |
| --- | --- |
| **Task (TK)** | As a result of these coaching sessions with my AI coach, I am clearer as to how I might be able to change. |
|  | What I am doing in coaching with my AI coach gives me new ways of looking at my problem. |
|  | I feel that the things I do in coaching with my AI coach will help me to accomplish the changes that I want. |
|  | I believe the way we worked together on my problem is correct. |
| **Bond (BD)** | I believe my AI coaching chatbot liked me. |
|  | My AI coaching chatbot and I respected each other. |
|  | I feel that my AI coaching chatbot appreciated me. |
|  | I feel my AI coaching chatbot cared about me even when I said things that he/she did not approve of. |
| **Goal (GL)** | My AI coaching chatbot and I collaborated on setting goals for my coaching. |
|  | My AI coaching chatbot and I worked towards mutually agreed-upon goals. |
|  | My AI coaching chatbot and I agreed on what is important for me to work on. |
|  | My AI coaching chatbot and I established a good understanding of the kind of changes that would be good for me. |

A 5-point Likert scale was used to measure agreement with the statements.
